# Meeting tomorrow’s needs: a single-centre study in geriatric neurosurgery

**DOI:** 10.1186/s12877-025-06489-1

**Published:** 2025-10-29

**Authors:** Einar Naveen Møen, Stephanie Schipmann, Rupavathana Mahesparan

**Affiliations:** 1 Department of Clinical Medicine, Faculty of Medicine, University of Bergen, Bergen, Norway; 2 Department of Neurosurgery, Haukeland University Hospital, Bergen, Norway; 3 Faculty of Medicine, University Hospital Münster, Münster, Germany

**Keywords:** Geriatric neurosurgery, Choosing wisely, Surgery duration, Health service planning, Emergency admissions, Frailty assessment

## Abstract

**Background:**

The world is experiencing a demographic shift towards a larger proportion of older people. This has important consequences for the healthcare system, including neurosurgical departments. Geriatric patients represent a range of unique challenges related to treatment, resource allocation, prioritization and ethical concerns. In order to meet the challenge of increasing demand of geriatric neurosurgical care, and prepare for planning and establishment of health service requirements, we must first understand the demographic trends, common diagnoses, and types of procedures geriatric patients undergo. The objective of this study is to investigate and report these parameters.

**Methods:**

Data was retrieved from the Lifecare Orbit Surgical Management system on all geriatric patients (≥ 65) treated at Haukeland University Hospital from January 1, 2018, to December 31, 2023. The dataset included date of birth, date of surgery, duration of surgery, procedure codes, diagnosis codes, and type of admission. Descriptive statistics and linear regression were performed using the R programming language.

**Results:**

We identified 2,512 patients eligible for inclusion. The most common procedures in our geriatric population were decompression of lumbar spinal canal and nerve roots (8.54%), decompression of lumbar nerve roots (7.38%), and evacuation of subdural hematoma (7.16%). The prevalence of emergency procedures increased with advancing age, and conversely, more complex elective procedures decreased with advancing age.

**Conclusions:**

We identified a shift towards emergency-driven care and a reduction in complex procedures as patients age. Further analysis of resource utilization and clinical outcomes, such as length of stay, will be essential in guiding future strategies for geriatric neurosurgical care.

**Supplementary Information:**

The online version contains supplementary material available at 10.1186/s12877-025-06489-1.

## Introduction

The relevance of geriatric neurosurgery is increasing. Geriatric patients, conventionally defined as those over and including 65 years, have increased rapidly and will continue to grow. By 2050, the number of people aged 60 and above is expected to double to 2.1 billion, while those aged 80 and above will triple, reaching 426 million [1]. This trend is also representative of Norway, where the percentage of older people is expected to increase from 13 percent today to 22 percent in 2060 [2]. Geriatric patients in neurosurgery represent a range of unique challenges, including those related to treatment, resource allocation, prioritization and ethical concerns [3, 4]. 

Considering that the challenges associated with older patients are expected to grow in importance, significance and magnitude, there is a concerning lack of tools for risk–benefit assessments, ethical considerations, and frameworks for resource allocation and prioritization. Moreover, descriptive studies on the most common procedures and diagnoses in geriatric neurosurgery are scarce. In order to meet these challenges, we must first understand the demographic trends, common diagnoses, and types of procedures geriatric patients undergo.

Addressing these gaps will not only improve patient outcomes, but also inform best practices and policies in geriatric neurosurgery. Thus, the objective of this study is to analyse the demographic trends of geriatric patients, and the types of neurosurgical procedures performed at a tertiary referral centre in the last five years. Our analysis will include examining the frequency of emergency and planned surgeries, the duration of surgeries, type of surgeries, and identifying future service needs and areas for improvement.

## Methods

### Study design

We conducted a cross-sectional population-based study. Data were retrieved from the Lifecare Orbit Surgical Management system, including all surgically treated patients at the Department of Neurosurgery at Haukeland University Hospital between January 1, 2018, and December 31, 2023.

### Study setting

The Department of Neurosurgery at Haukeland University Hospital serves as the tertiary referral centre for approximately 1.1 million inhabitants in Western Norway, providing the full spectrum of neurosurgical care. With the exception of a limited number of spinal procedures performed at private facilities, all patients within the region in need of neurosurgical care are treated at this centre. Although Norway’s healthcare system allows for some degree of patient choice in elective procedures, this is very rarely exercised in neurosurgery due to the highly specialized nature of the field, the limited number of neurosurgical centres nationwide, and geographic factors such as proximity and travel distance.

### Material

The dataset included each patient’s Orbit identification number, the first six digits of their national identification number (ddmmyy), the date of surgery, duration of surgery, procedure codes (Nordic Medico-Statistical Committee Classification of Surgical Procedures, NOMESCO), diagnosis codes (International Classification of Diseases Version 10, ICD-10), age at the time of data extraction, and type of admission. We used an algorithm in Excel to calculate the patient’s complete birth date (Appendix 1).

### Statistical analysis

All statistical analyses were conducted using RStudio with the programming language R. The following libraries were utilized: readxl, dplyr, tidyr, lubridate, stringr, and ggplot2 [5–10]. Subsetting techniques were used to stratify the population by age groups and years, describing the number of patients in each group, type of admission (emergency or planned), and most common diagnoses and procedures. Linear regression was employed for statistical analysis. ChatGPT versions 4o and 1o-preview were consulted for debugging of code [11].

The relationship between age and emergency admission was assessed using linear regression. The proportion of emergency patients for each age served as the dependent variable, while age was used as an independent variable. The *p*-value for the slope coefficient was used to determine statistical significance. Similarly, trends in the number of geriatric patients over time were assessed with a linear regression analysis. The number of geriatric patients each year served as the dependent variable, and year as a continuous independent variable. The *p*-value for the slope coefficient was used to determine statistical significance.

The relationship between age and surgery duration was usinglinear regression with surgery duration as the dependent variable, and age at surgery as the independent variable. The *p*-value for the slope coefficient was used to evaluate significance. Additionally, we conducted separate analyses for the top nine most common procedures to identify possible procedure-specific differences. AAK10 (duraplasty) was excluded from this analysis as it was never done in isolation as an independent procedure. A detailed explanation of the codes of the surgical procedures (NOMESCO) and diagnoses (ICD-10) mentioned in the results is provided in the supplementary appendix (Appendix 2).

## Results

Between January 1, 2018, and December 31, 2023, a total of 11,686 patients underwent surgery at the Department of Neurosurgery at Haukeland University Hospital. We identified 2,512 patients over 65 years who were eligible for inclusion (Appendix 3). This constituted 31% of the population who had undergone invasive neurosurgery and had a retrievable date of birth. Table 1 presents the demographic distribution of geriatric neurosurgery patients in the study period, stratified by age group and year. Most patients were in the younger age groups, with 31.99% (803) patients aged 65–69, and 29.22% (734) patients aged 70–74. There was a significant decline in the number of patients as age increased (*p-value:* 0.0038), with notably fewer individuals in the oldest age groups of 85 to 89 and ≥ 90. (Appendix 4).

**Table 1:** Demographics of geriatric neurosurgery patients by year

		2018	2019	2020	2021	2022	2023	Total
*Age 65—69*								
*Patients*	*n*	121	157	125	149	126	125	803
*Emergency*	*%*	45,45%	49,68%	40,80%	46,98%	34,13%	52,00%	45,08%
*Procedures*	*n*	144	241	191	224	196	209	1205
*Diagnoses*	*n*	94	96	84	126	96	105	601
Age 70–74								
*Patients*	*n*	126	129	130	120	109	120	734
*Emergency*	*%*	52,38%	39,53%	53,08%	40,00%	34,86%	42,50%	44,01%
*Procedures*	*n*	161	177	178	183	172	185	1056
*Diagnoses*	*n*	89	101	94	92	93	103	572
Age 75–79								
*Patients*	*n*	96	88	109	82	96	104	575
*Emergency*	*%*	43,75%	44,32%	52,29%	46,34%	41,67%	37,50%	44,35%
*Procedures*	*n*	113	111	163	110	135	151	783
*Diagnoses*	*n*	74	62	75	56	80	89	436
Age 80–84								
*Patients*	*n*	35	48	53	54	39	51	280
*Emergency*	*%*	57,14%	45,83%	62,26%	51,85%	48,72%	47,06%	52,14%
*Procedures*	*n*	43	56	78	70	49	64	360
*Diagnoses*	*n*	24	36	31	40	33	51	215
Age 85–89								
*Patients*	*n*	13	18	18	21	13	18	101
*Emergency*	*%*	69,23%	72,22%	55,56%	38,10%	46,15%	55,56%	55,45%
*Procedures*	*n*	14	25	20	25	17	23	124
*Diagnoses*	*n*	12	9	13	15	12	18	79
Age ≥ 90								
*Patients*	*n*	5	3	2	2	2	5	19
*Emergency*	*%*	80,00%	100,00%	50,00%	100,00%	100,00%	100,00%	89,47%
*Procedures*	*n*	5	3	2	2	2	6	20
*Diagnoses*	*n*	5	1	2	1	2	5	16

Legend: This table presents the number of patients, percentage of emergency patients (where the rest are elective patients), number of procedures, and number of diagnoses for each geriatric age group every year from January 1, 2018, to December 31, 2023, and as a five-year total, at Haukeland University Hospital, Department of Neurosurgery

**Fig. 1:**
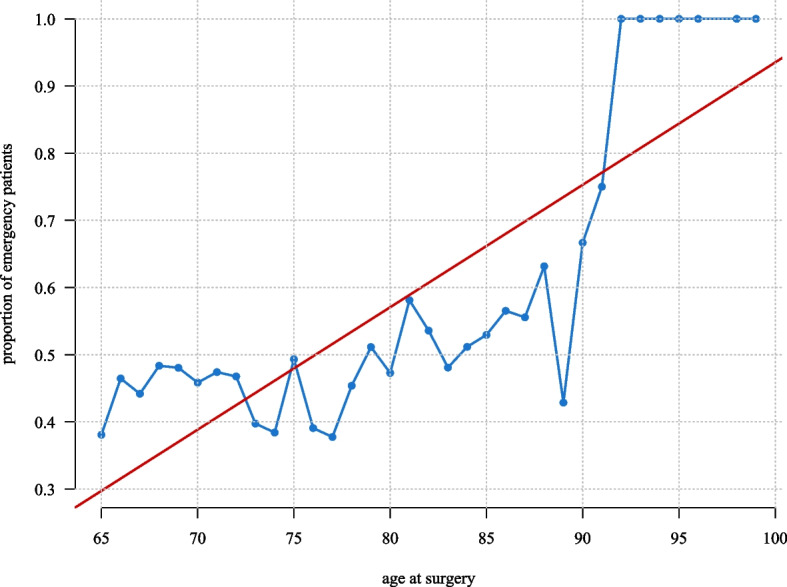
Average proportion of geriatric emergency patients by age. Legend: The blue line shows the proportion treated as emergency patients by age at Haukeland University Hospital, Department of Neurosurgery, between January 1, 2018, and December 31, 2023. The red line is a linear regression line predicting the relationship between proportion of emergency patients and age at surgery (*p*-value: < 0.001)

The number of procedures and diagnoses followed a similar pattern, with higher procedure counts in the younger age groups. Emergency procedures were proportionally higher among older age groups (Fig. 1). We found a significant positive association between age at surgery and proportion of emergency patients (*p*-value: < 0.0010). No significant trend was found forthe number of geriatric patients for each year in the study period (Appendix 5).

**Fig. 2:**
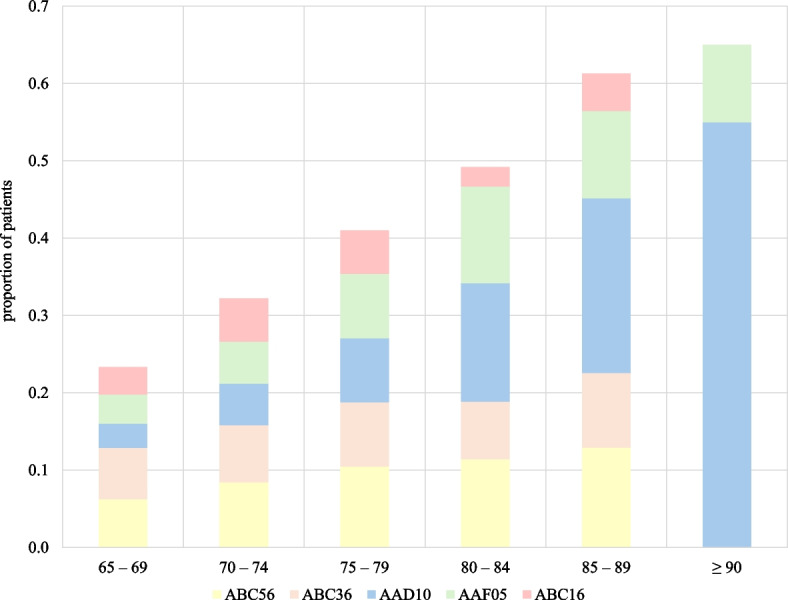
Change in top five procedures by age group. Legend: This figure shows how the top 5 most common surgeries in the entire geriatric population (≥ 65) treated at the Department of Neurosurgery, Haukeland University Hospital, between January 1, 2018, and December 31, 2023, changes in different age groups. Code ABC56 defines decompression of lumbar spinal canal and nerve roots. Code ABC36 defines decompression of lumbar nerve roots. Code AAD10 defines evacuation of subdural hematoma. Code AAF05 defines ventriculoperitoneal shunting. Finally, code ABC16 defines excision of lumbar intervertebral disc displacement

We identified the ten most frequent neurosurgical procedures in the geriatric population using codes from NOMESCO (Appendix 6). Microsurgical excision of lumbar intervertebral disc displacement (ABC16) was the most frequent procedure in the 65–69 years group (7.22%). Resection of intracranial lesions (AAB10) was the second most frequent procedure in this age group (6.80%). Notably, both decreased in prevalence with advancing age. The most frequent procedure in the 70–74 and 75–79 years group was decompression of lumbar spinal canal and nerve roots (ABC56) (8.43% and 10.47%, respectively). Evacuation of subdural hematoma (AAD10) was the most frequent procedure in the 80—84 years group (15.28%) and above, and the third most frequent procedure in total in the geriatric population, reaching a prevalence of 55% in patients aged 90 and above (Fig. 2). The most common procedures in the geriatric population in total were decompression of lumbar spinal canal and nerve roots (ABC56) (8.54%), decompression of lumbar nerve roots (ABC36) (7.38%), and evacuation of subdural hematoma (AAD10) (7.16%). In comparison, the most frequent procedures in adults aged 18–64 were microsurgical excision of lumbar intervertebral disc herniation (ABC16) (14.97%), anterior decompression of cervical spine with insertion of interbody fixating implant (ABC21) (9.07%), and decompression of lumbar nerve roots (ABC36) (7.93%) were the three most prevalent procedures.

Table 2 presents the ten most frequent neurosurgical diagnoses in the geriatric population using ICD-10 codes. Spinal stenosis (M48.0) was the most common diagnosis in the entire geriatric population with a prevalence of 14.43%, followed by lumbar and other disc disorders with myelopathy (M51.1) and traumatic subdural haemorrhage (S06.50), with prevalences of 9.38% and 7.61%, respectively. We found that spinal stenosis (M48.0) was the most prevalent diagnosis in the 65–69 (12.48%), peaking in the 75–79 and 80–84 groups (16.28%). Notably, S06.50 steadily increased in prevalence with advancing age, becoming the most common diagnosis in the 85–89 and ≥ 90 groups, with prevalences of 20.25% and 56.25% respectively. We also observed treatment for malignant neoplasms in the cerebrum (C71.0) decreased with age, while nontraumatic subdural haemorrhage (I62.0), and communicating (G91.0), obstructive (G91.1) and normal-pressure hydrocephalus (G91.3) increased with age. In comparison, the three most prevalent diagnoses in the 18–64 cohort was lumbar and other intervertebral disc disorders with radiculopathy (M51.1) with a prevalence of 27.20%, cervical disc disorder with myelopathy (M50.1) at 14.04%, and C71.0 at 5.18%.

**Table 2:** Most common geriatric neurosurgical diagnoses (ICD-10)

**#**		**65–69**	**70–74**	**75–79**	**80–84**	**85–89**	** ≥ 90**	** ≥ 65**	**18–64**
**1**	*diagnosis*	M480	M480	M480	M480	S0650	S0650	M480	M511
	*n*	75	83	71	35	16	9	277	1025
	*%*	12,48%	14,51%	16,28%	16,28%	20,25%	56,25%	14,43%	27,20%
**2**	*diagnosis*	M511	M511	M511	S0650	I620	I620	M511	M501
	*n*	69	53	40	32	14	3	180	529
	*%*	11,48%	9,27%	9,17%	14,88%	17,72%	18,75%	9,38%	14,04%
**3**	*diagnosis*	I671	D320	S0650	G912	M480	G912	S0650	C710
	*n*	44	45	38	24	13	2	146	195
	*%*	7,32%	7,87%	8,72%	11,16%	16,46%	12,50%	7,61%	5,18%
**4**	*diagnosis*	D320	G910	G912	I620	G912	D352	D320	D320
	*n*	35	33	33	14	9	1	101	170
	*%*	5,82%	5,77%	7,57%	6,51%	11,39%	6,25%	5,26%	4,51%
**5**	*diagnosis*	I609	S0650	C710	G910	M511	S010	C710	I671
	*n*	34	32	30	13	7	1	96	148
	*%*	5,66%	5,59%	6,88%	6,05%	8,86%	6,25%	5,00%	3,93%
**6**	*diagnosis*	C710	C710	I620	M511	D320	*NA*	G912	M480
	*n*	33	29	29	11	3	*NA*	96	142
	*%*	5,49%	5,07%	6,65%	5,12%	3,80%	*NA*	5,00%	3,77%
**7**	*diagnosis*	D352	I609	G911	G911	D339	*NA*	I620	I609
	*n*	25	27	20	8	2	*NA*	96	127
	*%*	4,16%	4,72%	4,59%	3,72%	2,53%	*NA*	5,00%	3,37%
**8**	*diagnosis*	G910	G911	D352	I609	G910	*NA*	G910	D352
	*n*	23	23	19	7	2	*NA*	90	124
	*%*	3,83%	4,02%	4,36%	3,26%	2,53%	*NA*	4,69%	3,29%
**9**	*diagnosis*	M501	I620	G910	T850	I609	*NA*	I609	D333
	*n*	22	23	19	6	2	*NA*	80	112
	*%*	3,66%	4,02%	4,36%	2,79%	2,53%	*NA*	4,17%	2,97%
**10**	*diagnosis*	S0650	I671	D320	D320	D329	*NA*	I671	G911
	*n*	19	19	13	5	1	*NA*	70	103
	*%*	3,16%	3,32%	2,98%	2,33%	1,27%	*NA*	3,65%	2,73%

Legend: This table presents the ten most common neurosurgical diagnoses (using the ICD-10 classification) in geriatric patients at Haukeland University Hospital in the period January 1, 2018, to December 31, 2023, for each age group and in total. The data is presented as both absolute numbers (*n*) and percentage (%) of the total number of diagnoses. For comparison, the table also presents the top ten most common neurosurgical diagnoses in adults aged from and including 18 to 65.

**Fig. 3:**
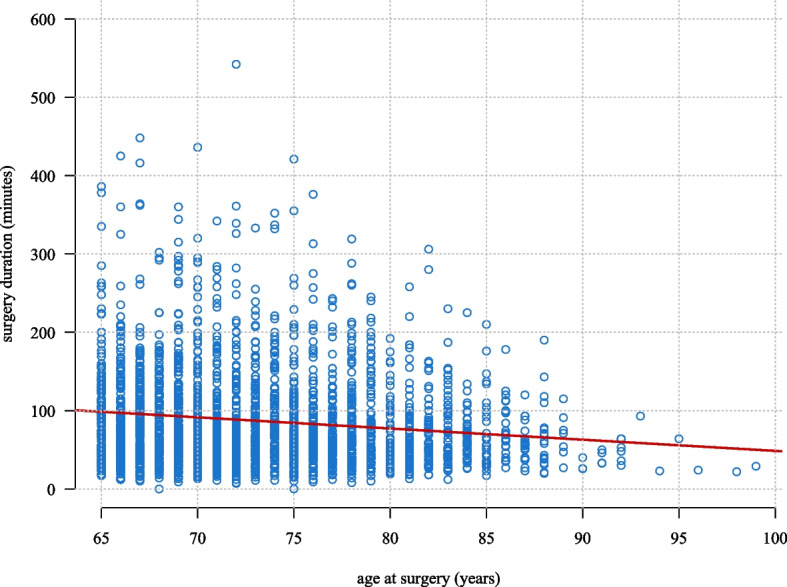
Duration of Surgery by Age in Total. Legend: This figure shows the duration of surgery in minutes by age at surgery for geriatric patients at Haukeland University Hospital, Department of Neurosurgery, between the dates of January 1, 2018, and December 31, 2023. Every blue point represents one patient with a corresponding age and surgery duration. The red line is a regression line predicting the relationship between age at surgery and surgery duration (*p*-value: < 0.001)

Figure 3 presents the duration of surgery by age, unadjusted for type of procedure performed in the surgery. A strong association was observed between increased age and reduced surgery duration (*p*-value: < 0.0010). To account for differences in procedures, as we observed that older patients are rarely selected for complex procedures, we analysed surgery duration by age within the nine most frequent procedures. We found a significant positive association between age and surgery duration for microsurgical excision of lumbar intervertebral disc displacement (ABC16) (*p*-value: < 0.0010). No other statistically significant relationships were identified across the remaining eight procedures (Appendix 7).

## Discussion

### Type of procedures

We observed a decline in the overall number of procedures performed with advancing patient age. The procedural and diagnostic spectrum also narrowed, with a clear trend towards greater homogeneity. Notably, the proportion of emergency admissions increased substantially with age, reflecting a shift from elective to acute care in the oldest patients—a pattern particularly pronounced in those over 80 years of age. This transition appears to be a unique characteristic of the geriatric neurosurgical population at our centre. Additionally, we observed a significant decline in complex and resource-intensive interventions, such as resection of intracranial lesions (AAB10), as patient age increased.

### Age and surgery duration

When analysing the nine most common procedures in our data, we found no significant correlation between age and surgery duration, except for microsurgical excision of lumbar intervertebral disc displacement (ABC16). Considering surgery duration as an indicator of intraoperative complications and difficulty, this indicates that age may not necessarily be a risk factor, given that the patient is deemed fit for surgery. However, the overall trend towards fewer complex surgeries and shorter procedures might reflect a clinical caution. As patients age, clinicians might be more reserved in recommending complex surgeries due to concerns about frailty and comorbidities. Given the evidence of improved health outcomes in older populations [12–14], it is possible that some otherwise healthy older patients are not receiving potentially beneficial surgeries because of their age alone.

### Comparison of findings

To our knowledge, the only similar study to date was conducted in 1987 using patient data from the Department of Neurosurgery at Henry Ford Hospital between January 1, 1978, and January 1, 1985 [15]. Interestingly, our findings share several similarities with the results of that study. In the 1987 study, geriatric patients constituted 20.20% of their population, whereas we found a higher proportion at 32.70%. The average geriatric patient age in their material was 72 years old, whereas we found a mean age of 73.80 years. Furthermore, they identified occlusive cerebrovascular disease, spinal degenerative myeloradiculopathy, and tumours as the most frequent diseases in their population. We found spinal stenosis (M48.0), lumbar and other intervertebral disc disorders with radiculopathy (M51.1), and traumatic subdural haemorrhage (S06.50) as the three most frequent diagnoses in the entire geriatric population.

### Strengths and limitations

There are several limitations to our study. The most important limitation is the uncertainty regarding the generalizability of our findings considering differences in population and health demographics. However, they are likely most relevant to neurosurgical centres in Scandinavia and Northern Europe, where population characteristics and publicly funded healthcare systems—such as Norway’s universal, tax-based model offering care at no direct cost to patients—are broadly comparable. Information bias represents another limitation, as 1,179 patients with incomplete data were excluded. We estimate that one-fourth (295) to one-third (393) of this cohort are geriatric patients. This exclusion could lead to an underrepresentation of certain procedures and diagnoses. Furthermore, our study period of five years is likely insufficient to reflect long-term demographic patterns. There is also an unexplained decrease in geriatric patients between 2022 and 2023, possibly related to disruptions from the COVID-19 pandemic. Unmeasured confounding variables could have affected our statistical analyses. While unlikely, we recognize that there is a potential for age misclassification from our birth date algorithm (Appendix 1), representing an additional limitation.

Despite these limitations, our study provides real-world data and insight on geriatric neurosurgical characteristics from a representative regional neurosurgical centre with the whole spectrum of neurosurgical care for all age groups thereby minimizing patient driven selection bias and pathology-specific selection bias. Additionally, the findings in our study share similarities with those identified in the comparable study from Henry Ford Hospital, suggesting some core patterns in geriatric neurosurgery that transcends healthcare system differences. Collectively, the patterns, trends and characteristics identified in this paper do provide practical insights that can be used for healthcare planning and resource allocation.

### Implications

At our department, we already perform thorough patient selection for elective surgeries based on general health, comorbidities, expected outcomes, life expectancy, patient preferences, surgical risk, and rehabilitation potential. However, we currently do not apply specific scoring systems tailored for older patients. The decline in more complex surgeries, coupled with the observed decrease in overall surgery duration with age, raises an important point. Are we choosing wisely, or could this represent a potential undertreatment of older patients [16]? This calls for a discussion about the appropriate selection criteria for neurosurgery in older patients, with a focus on screening of frailty, as well as physiological and neurological reserve capacity [3, 17–19].

## Conclusion

This study provides insights into characteristics and trends within the geriatric neurosurgical population in a real-world situation at our department. Almost one-third of our patient population are geriatric patients. We found a shift toward emergency-driven care and a reduction in complex procedures as patients age. Moving forward, a more nuanced approach to patient selection incorporating frailty assessments will be crucial to ensure optimal care for the growing population of older people. Further analysis of resource utilization and clinical outcomes, such as length of stay, is warranted and will be addressed in an on-going study from our department.

## Supplementary Information

Supplementary Material 1.

## Data Availability

The datasets generated and analyzed during the current study are not publicly available due to the inclusion of confidential information of the included patients.
