# Supplementary material for: Meeting tomorrow’s needs: a single-centre study in geriatric neurosurgery
Source: BMC Geriatr. 2025 Oct 29;25:821. doi: 10.1186/s12877-025-06489-1 (PMC12573956; doi:10.1186/s12877-025-06489-1)
Supplement: Supplementary file 1 — Supplementary Material 1. [file 12877_2025_6489_MOESM1_ESM.docx]

# Appendix 1: Explanation of Algorithm

Algorithm 1 calculated the patient’s birth year based on their age at the time of data extraction. The algorithm initially assumed the patient was born in the 20^th^ century and calculated the corresponding age. If this age was not close to the age given at the extraction, the algorithm inferred that the patient was born in the 21^st^ century. Conversely, if the age matched, the patient was confirmed to have been born in the 20^th^ century.

| Appendix 2: Explanation of Mentioned Diagnoses and Procedures | | | |
| --- | --- | --- | --- |
| Diagnoses (ICD-10) | | Procedures (NOMESCO) | |
| Code | Explanation | Code | Explanation |
| I609 | unspecified subarachnoid hemorrhage | AAF20 | revision of shunt of ventricle of brain |
| C79.3 | secondary malignant neoplasm of brain and cerebral meninges | NAG70 | interlaminary fusion of cervical spine with fixation |
| T85.0 | mechanical complication of ventricular intracranial shunt | AAD11 | evacuation of chronic subdural hematoma with burr hole |
| D33.9 | benign neoplasm in unspecified part of central nervous system | AAD12 | evacuation of chronic subdural hematoma with craniotomy |
| G20 | parkinson's disease | AAD05 | evacuation of acute subdural hematoma |
| S01.0 | open wound of scalp | AAE10 | transsphenoidal total or partial excision of intracranial lesion |
| S06.5 | traumatic subdural hemorrhage | QAB05 | revision of wound of skin of head or neck |
| D43.0 | neoplasm of uncertain or unknown behavior | ABC60 | decompression of cervical spinal cord |
| G93.1 | anoxic brain damage | AAD10 | evacuation of chronic subdural hematoma |
| M50.0 | cervical disc disorder with myelopathy | AAF05 | ventriculoperitoneal shunt |
| I62.0 | nontraumatic subdural hemorrhage | ABC16 | microsurgical excision of lumbar intervertebral disc displacement |
| G91.0 | communicating hydrocephalus | ABC21 | anterior decompression of cervical spine with insertion of interbody fixating implant |
| G91.2 | normal-pressure hydrocephalus | ABC36 | decompression of lumbar nerve roots |
| M51.1 | lumbar and other intervertebral disc disorders with radiculopathy | AAB10 | microsurgical excision of cervical intervertebral disc displacement |
| M50.1 | cervical disc disorder with radiculopathy | ABC26 | open discectomy of lumbar spine |
| C71.0 | malignant neoplasm of cerebrum, except lobes and ventricles | AAK10 | repair of dura |
| D32.0 | benign neoplasm of cerebral meninges | AAB00 | extirpation of intracranial lesion |
| I67.1 | cerebral aneurysm, unruptured | AAY00B | endovascular occlusion of intracranial aneurism |
| M48.0 | spinal stenosis | ABC56 | decompression of lumbar spinal cord and nerve roots |
| I60.9 | subarachnoid hemorrhage, unspecified | AAF02 | external drainage of ventricle of brain |
| D35.2 | benign neoplasm, pituitary gland |  |  |
| D33.3 | benign neoplasm, cranial nerves |  |  |
| G91.1 | obstructive hydrocephalus |  |  |

*Appendix 1. This table contains explanations of the mentioned procedures (NOMESCO) and diagnoses (ICD-10) in our study.*

# Appendix 3: Flow Chart of Inclusion Process

Total surgeries

n = 11,686

Error in national ID

n = 35
Date of birth not retrievable

n = 1,179
Non-invasive neurosurgery

n = 2,369

Non-geriatric patients

n = 5,591

Excluded

n = 9,174

Included

n = 2,512

*Appendix 2. This figure shows the process of inclusion in our retrospective analysis.*

# Appendix 4: Proportion of Geriatric Patients by Age Group


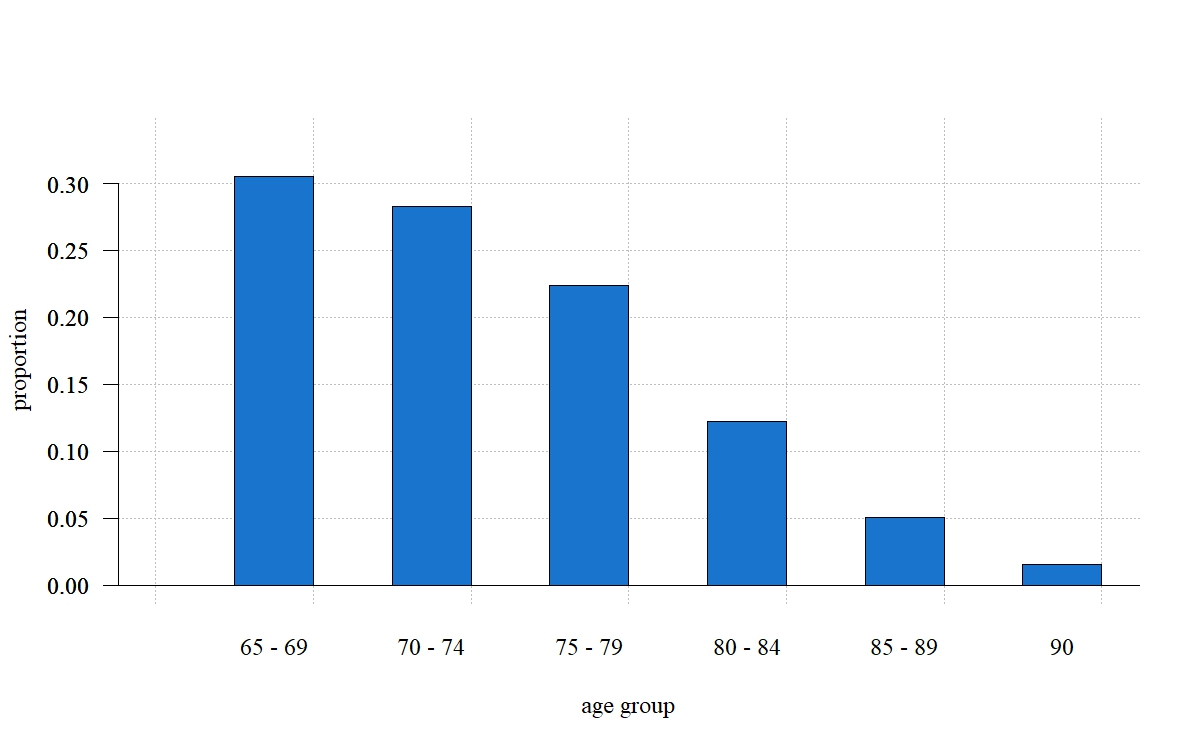


*Appendix 3. This bar plot shows the age distribution of geriatric patients treated at Haukeland University Hospital, Department of Neurosurgery, between 1^st^ January 2018 and 31^st^ December 2023. Each bar represents the proportion of geriatric patients in each age group by the total number of geriatric patients. The p-value for the slope coefficient was 0.00038.*

# Appendix 5: Number of Geriatric Patients by Year


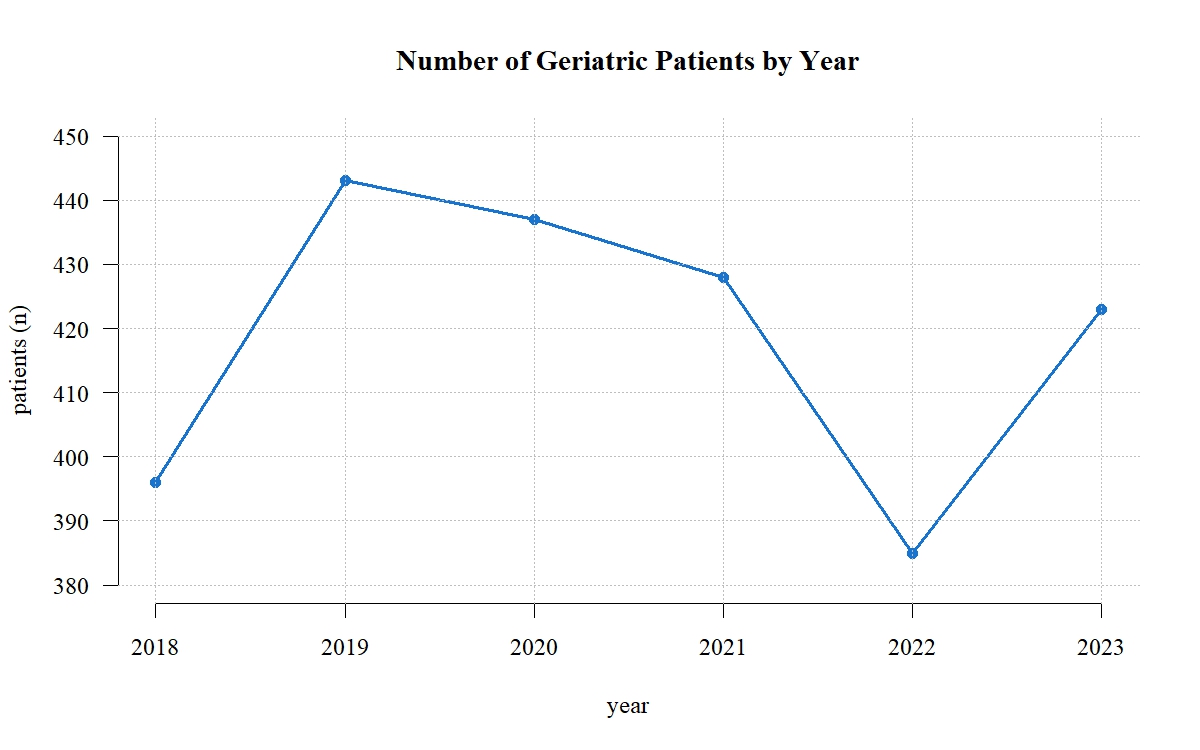


*Appendix 4. The blue line shows the total number of geriatric patients each year at Haukeland University Hospital, Department of Neurosurgery, between 1^st^ January 2018 and 31^st^ December 2023.*

| Appendix 6: Most Common Geriatric Neurosurgical Procedures (NOMESCO) | | | | | | | | | |  |
| --- | --- | --- | --- | --- | --- | --- | --- | --- | --- | --- |
| **#** |  | 65 – 69 | 70 – 74 | 75 – 79 | 80 – 84 | 85 – 89 | ≥ 90 | ≥ 65 | 18 - 64 | |
| **1** | *procedure* | ABC16 | ABC56 | ABC56 | AAD10 | AAD10 | AAD10 | ABC56 | ABC16 | |
|  | *n* | 87 | 89 | 82 | 55 | 28 | 11 | 303 | 1284 | |
|  | *%* | 7,22 % | 8,43 % | 10,47 % | 15,28 % | 22,58 % | 55,00 % | 8,54 % | 14,97 % | |
| **2** | *procedure* | AAB10 | ABC36 | AAD10 | AAF05 | ABC56 | AAD11 | ABC36 | ABC21 | |
|  | *n* | 82 | 78 | 65 | 45 | 16 | 3 | 262 | 778 | |
|  | *%* | 6,80 % | 7,39 % | 8,30 % | 12,50 % | 12,90 % | 15,00 % | 7,38 % | 9,07 % | |
| **3** | *procedure* | ABC36 | AAK10 | AAF05 | ABC56 | AAF05 | AAD12 | AAD10 | ABC36 | |
|  | *n* | 80 | 68 | 65 | 41 | 14 | 2 | 254 | 680 | |
|  | *%* | 6,64 % | 6,44 % | 8,30 % | 11,39 % | 11,29 % | 10,00 % | 7,16 % | 7,93 % | |
| **4** | *procedure* | ABC56 | AAB10 | ABC36 | ABC36 | ABC36 | AAF05 | AAF05 | ABC26 | |
|  | *n* | 75 | 64 | 65 | 27 | 12 | 2 | 228 | 532 | |
|  | *%* | 6,22 % | 6,06 % | 8,30 % | 7,50 % | 9,68 % | 10,00 % | 6,43 % | 6,20 % | |
| **5** | *procedure* | AAK10 | ABC16 | AAB10 | AAF20 | ABC16 | AAE10 | ABC16 | AAB10 | |
|  | *n* | 73 | 59 | 44 | 12 | 6 | 1 | 205 | 479 | |
|  | *%* | 6,06 % | 5,59 % | 5,62 % | 3,33 % | 4,84 % | 5,00 % | 5,78 % | 5,58 % | |
| **6** | *procedure* | AAY00B | AAD10 | ABC16 | AAF02 | AAD11 | QAB05 | AAB10 | AAK10 | |
|  | *n* | 55 | 57 | 44 | 10 | 5 | 1 | 200 | 370 | |
|  | *%* | 4,56 % | 5,40 % | 5,62 % | 2,78 % | 4,03 % | 5,00 % | 5,64 % | 4,31 % | |
| **7** | *procedure* | AAF02 | AAF05 | AAK10 | AAK10 | AAB00 | *NA* | AAK10 | AAB00 | |
|  | *n* | 50 | 57 | 34 | 10 | 3 | *NA* | 188 | 272 | |
|  | *%* | 4,15 % | 5,40 % | 4,34 % | 2,78 % | 2,42 % | *NA* | 5,30 % | 3,17 % | |
| **8** | *procedure* | AAB00 | AAB00 | AAF02 | AAB10 | AAK10 | *NA* | AAB00 | AAY00B | |
|  | *n* | 45 | 54 | 25 | 9 | 3 | *NA* | 129 | 242 | |
|  | *%* | 3,73 % | 5,11 % | 3,19 % | 2,50 % | 2,42 % | *NA* | 3,64 % | 2,82 % | |
| **9** | *procedure* | AAF05 | AAF02 | AAB00 | ABC16 | AAY00B | *NA* | AAF02 | ABC56 | |
|  | *n* | 45 | 40 | 23 | 9 | 3 | *NA* | 127 | 211 | |
|  | *%* | 3,73 % | 3,79 % | 2,94 % | 2,50 % | 2,42 % | *NA* | 3,58 % | 2,46 % | |
| **10** | *procedure* | ABC21 | AAY00B | AAE10 | ABC60 | NAG70 | *NA* | AAY00B | AAF02 | |
|  | *n* | 43 | 32 | 18 | 8 | 3 | *NA* | 110 | 183 | |
|  | *%* | 3,57 % | 3,03 % | 2,30 % | 2,22 % | 2,42 % | *NA* | 3,10 % | 2,13 % | |

*Table 2. This table presents the ten most common neurosurgical procedures (using the NOMESCO classification of surgical procedures) in geriatric patients at Haukeland University Hospital in the period 1^st^ January 2018 to 31^st^ December 2023 per age group and in total. The data is presented as both absolute number of procedures (n) and percentage (%) of the total number of procedures. For comparison, the table also presents the top ten most common neurosurgical procedures in adults aged 18 to 65.*

# Appendix 7: Duration of Surgery by Age in Individual Surgeries


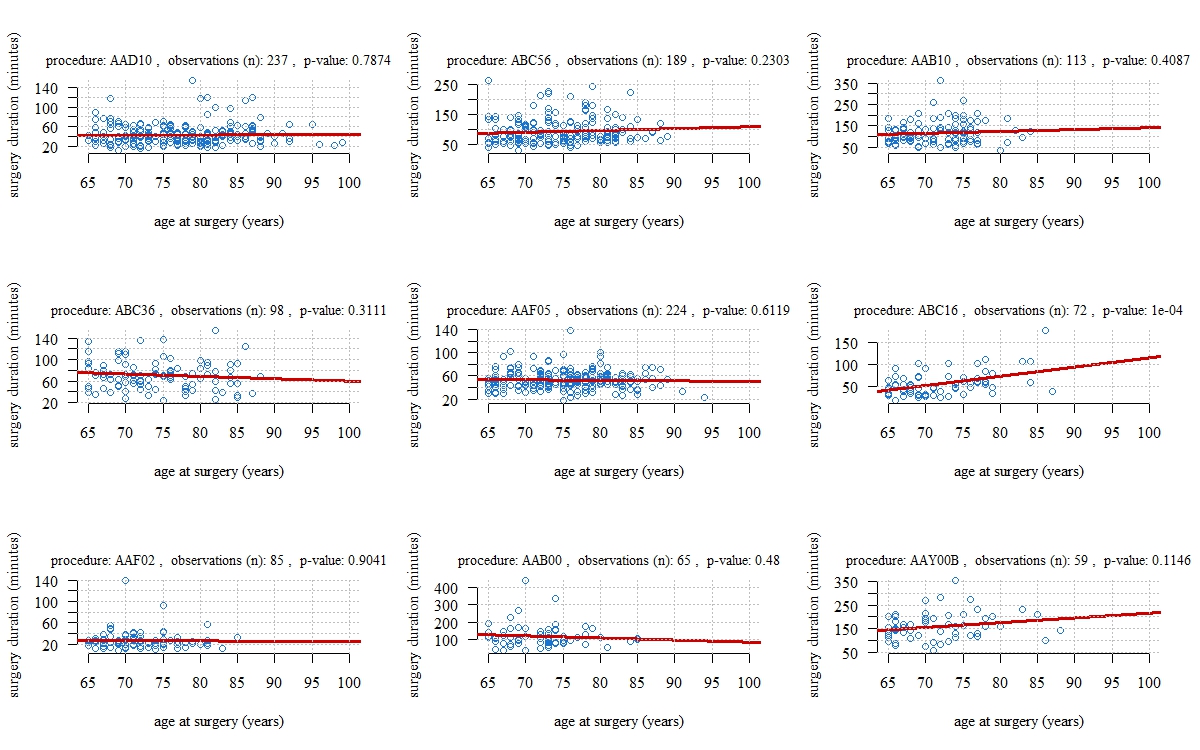


*Appendix 6. This figure shows the duration of surgery in minutes by age for the nine most common procedures in geriatric patients, except AAK10, for all patients at Haukeland University Hospital, Department of Neurosurgery, between the dates of 1^st^ January 2018 and 31^st^ December 2023. Every blue point represents one patient with a corresponding age and surgery duration. The red line is a regression line predicting the relationship between age at surgery and surgery duration for each surgery.*
